# Maternal *Lactobacillus rhamnosus* administration impacts neonatal CD4 T-cell activation and prevents murine T helper 2-type allergic airways disease

**DOI:** 10.3389/fimmu.2022.1082648

**Published:** 2023-01-04

**Authors:** Justine Smout, Clara Valentin, Sandrine Delbauve, Jeanne Pauwels, Arnaud Köhler, Véronique Flamand

**Affiliations:** ^1^Institute for Medical Immunology, Université Libre de Bruxelles, Gosselies, Belgium; ^2^ULB Center for Research in Immunology (U-CRI), Université Libre de Bruxelles, Gosselies, Belgium

**Keywords:** allergy, *Lactobacillus rhamnosus*, neonates, dendritic cells, CD4 T cells

## Abstract

Gut microbiota plays a role in the neonatal immune education and could influence susceptibility to Th2-type immune disorders, such as allergies, the most prevalent chronic diseases in early childhood. We studied the impact of oral *Lactobacillus rhamnosus* (*L.rhamnosus*) supplementation to pregnant/breastfeeding C57BL/6 mice on the development of allergic airways disease in their offspring. We observed that mice, from *L.rhamnosus*-treated mothers, inoculated with ovalbumin (OVA)-Aluminium hydroxide (ALUM) at 3 days of life and challenged intranasally 4 weeks later showed decreased Th2-associated cytokines, IgE and IgG1, lung eosinophilia and airway hyper-reactivity compared to OVA-sensitized mice from untreated mothers. In that setting, the *L.rhamnosus* treatment increased the number and maturation of splenic neonatal type 1 conventional dendritic cells (cDC1) that remained largely dominant over the cDC2 and favored their OVA-specific Th1 differentiation. In response to inhaled house dust mite (HDM) allergen, the maternal *L.rhamnosus* supplementation increased the number of neonatal pulmonary cDC1 expressing lower amount of costimulatory molecules compared with no supplementation and decreased the number of cDC2 without affecting their costimulatory molecules expression. An HDM-specific Foxp3^+^RORγt^+^ Treg polarization was monitored in the lung draining lymph nodes. Finally, we confirmed the inhibitory effect of maternal *L.rhamnosus* treatment on all the measured features of the HDM allergic airways reaction in their offspring. We conclude that maternal *L.rhamnosus* administration prevents Th2-type allergic airways disease in their neonates by favoring splenic cDC1/Th1 responses against ALUM-adjuvanted OVA or by promoting a pulmonary Foxp3^+^RORγt^+^ Treg activation against inhaled HDM.

## Introduction

There is now sufficient evidence that the neonatal immune system and its responses are unique, defined as unbalanced against Th1-cells polarizing cytokines. This makes newborns more vulnerable to pathogenic infections with impaired immune responses to most vaccines. Moreover, excessive Th2-cells related inflammation is a hallmark of many pathologies in early life such as allergies (1, 2). One of the mechanisms underlying the bias of T-cell response towards the Th2 type rather than the Th1 type at this age can be deciphered at the level of conventional dendritic cells (cDC) which are specialized to sense the environment, to present environmental antigens and to educate the adaptive T-cell response. A lack of IL-12p70, the Th1-polarizing cytokine, produced by type 1 conventional dendritic cells (cDC1) in response to most TLR ligands account for the impaired neonatal Th1-type response (3, 4). During the neonatal period, defined as 7 days post-delivery in mice, a predominance of cDC1 subset outnumbered cDC2 both in the spleen and in the lung. In the spleen, we have previously shown that despite their lack of IL-12p70 secretion, cDC1 possess the unique capacity to produce the anti-inflammatory cytokine IL-10 that inhibits type 1 immune responses (5). Neonatal splenic cDC2 induce greater Th17 and regulatory T cells (Treg) differentiation than adult cDC2, suggesting increased tolerogenic potential of splenic cDC2 in early life (6). In the lung, neonatal cDC1 have been shown to be less effective than their adult counterparts to process and present antigen *via* MHCI or II with lower levels of costimulatory molecules (7). cDC2 in the developing lung exhibit intrinsic Th2 bias. Indeed, in early life, IL-33 induces OX40L expression on cDC2, which promotes Th2 skewing (8).

Asthma is one of the most common chronic inflammatory diseases of the airways affecting one in ten children worldwide. It is associated with airway hyperresponsiveness (AHR) and remodeling (9). Most of childhood-onset asthma is driven by type 2 immune response, without sufficient regulation by type 1 (Th1 cells) and type 3 (RORγt^+^ Foxp3^+^ Treg) responses, and can be defined by the high level of immunoglobulins E (IgE) in the serum and eosinophilic airway inflammation (10, 11).

It is now clear that the increased prevalence of atopic disease and asthma development since the 1950s can be explained by a lack of microbial exposure, particularly during the neonatal period (12). For instance, children growing up on a farm where they are more exposed to microbial compounds have a decreased risk of allergic disease in later childhood. The induction of a Toll-like receptor (TLR)-dependent innate immunity contributes to the less allergic anti-Th2 immune phenotype observed in these children (13). Furthermore, maternal farm exposure in pregnancy was considered crucial to modulate their offspring sensitivity to allergens. Indeed, cord blood mononuclear cells of offspring of farming mothers displayed an increase in the number and function of Treg cells associated with lower Th2 cytokine secretion in response to allergen compared with nonfarming mothers (14).

Recently, a lot of attention has focused on the first exposure to commensals that can modulate the development of the immune system during fetal and early postnatal life (15, 16). Although debated, there is evidence that gastrointestinal bacterial colonization very likely begins before birth with a strong contribution from the mother (17). Moreover, it was recently demonstrated that variance in heritable vaginal bacteria and functions shared between mother and infant pairs relate to maternal allergy status and early-life markers of allergy such as IgE (18). This strongly reinforces the impact of vertically transmitted maternal microbiota *in utero* or in the early post-natal period that may influence gut microbiome and immune developmental trajectories in infancy.

Therefore, the early life colonization coincides with a time-limited period during which the immune system is permissive to microbial instruction (19). This has led to the concept of “window of opportunity” to educate the immune system for the whole life (20). Knowing that the microbiota may quickly be modified by many factors such as diet (21), antibiotic or probiotics use with potential impact on host immune system, supplementation with probiotics has been used as a preventive or therapeutic strategy for allergic disorders. So far, despite evidence of successful modification of the delayed gut microbiota development in infants at high risk for asthma thanks to their supplementation with *Lactobacillus* probiotics, it was not associated with a lower risk of asthma development (22). However, supplementation given during the pre- and post-natal period seems more likely to play a role in the prevention of this disorder (23). However, despite such supplementation strategies are applied today to pregnant and breastfeeding mothers, little is known about the impact of probiotics on both systemic and mucosal immune systems in the offspring.

Here we evaluate the effect of microbial education with *Lactobacillus* strain supplementation on the neonatal systemic and mucosal immune systems and on their susceptibility to Th2-driven allergic asthma. We reported that exclusive maternal *Lactobacillus rhamnosus* (*L.rhamnosus*) supplementation during pregnancy and breastfeeding protects their offspring from Th2-type allergic airway disease with an impact on the neonatal cDC compartments and CD4 T-cell differentiation.

## Materials and methods

### Mice

C57BL/6 mice were purchased from ENVIGO (Zeist, Netherlands). IL-10^reporter/GFP^ mice on a C57BL/6 genetic background were kindly provided by Carl de Trez (Vrije Universiteit Brussel, Brussels). Mice were housed and bred in our specific pathogen-free animal facility in individually ventilated cages with a controlled day-night cycle and given food and water *ad libitum*. Age of neonatal mice is specified for each experiment. All experiments were approved by the institutional Animal Care and Local Use Committee.

### *In vivo* treatment

The 16^th^ day of gestation (i.e., 5 days before delivery), pregnant females were orally administered daily with *Lactobacillus rhamnosus* VES001 (*L.rhamnosus*; 2.10^8^ CFU (Vésale Pharma, Eghezée, Belgium)/100 µL of NaCl 0,9% (B.Braun, Melsungen, Deutschland). The treatment was established after a dose-response assessment and corresponds to the most common dose (22, 24). It is maintained until postnatal day (PND)3 at the rate of one daily gavage.

### Murine models of allergic airway disease

**Figure 1:**
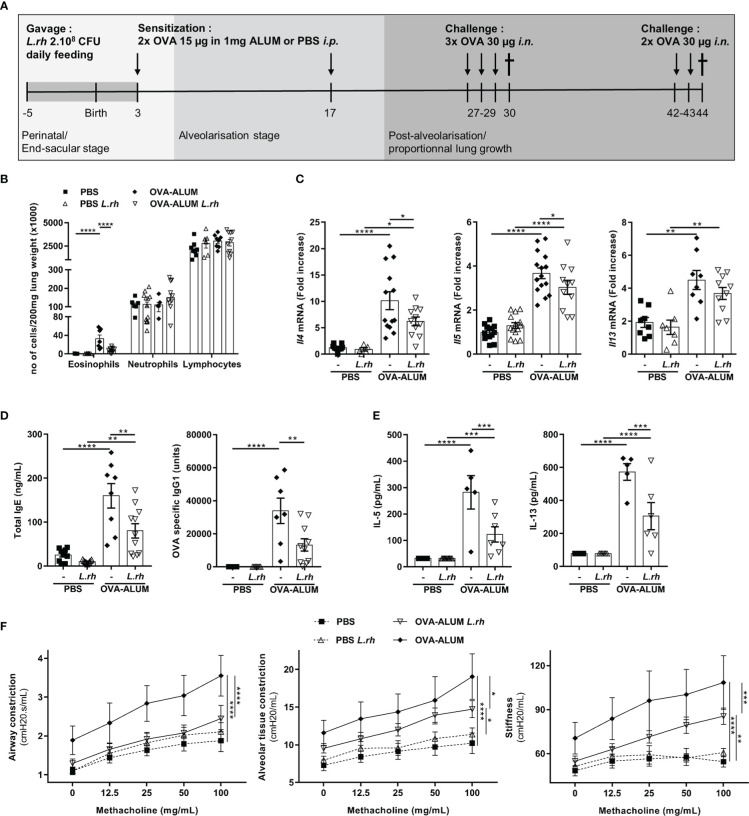
Perinatal *L.rhamnosus* exposures protects neonates from OVA-ALUM induced airway allergic reaction. **(A)** Protocol of airway allergic inflammation induction. Neonates born from C57BL/6 control (-) or probiotic-supplemented mother (*L.rh*) were mock-sensitized *i.p.* with PBS or with 15 µg OVA in 1 mg ALUM on postnatal day (PND) 3 and 17 and were challenged with 30 µg OVA on 3 consecutive days 10 days after the last sensitization. For flexiVent^©^ experiments, neonates were challenged again for 2 consecutive days with 30 µg OVA 2 weeks later. Mice were sacrificed on PND30 for the immunological analysis **(B–E)** and PND44 to assess the respiratory functions **(F)**. **(B)** Cell counts in the lung were determined 24h after the last challenge by flow cytometry (FACS). **(C)** Pulmonary mRNA normalized expression of *Il4*, *Il5* and *Il13* were analyzed by quantitative RT-PCR (qRT-PCR). Relative unit was obtained by comparing each group to the mean of non-treated PBS mice. **(D)** Total IgE and OVA-specific IgG1 in serum were quantified by ELISA. **(E)** Th2-associated cytokines in MLN restimulated for 3 days with 100 µg/mL OVA grade V were measured by ELISA. **(F)** AHR in response to increasing doses of methacholine was analyzed by flexiVent^©^ system. Results are a pool of 3–4 independent experiments and are shown as mean ± SEM. Statistical analysis was done using an ordinary one-way ANOVA (Holm-Sidak’s multiple comparisons test) or a Kruskal-Wallis test (Dunn’s multiple comparisons test) (* = p <0.05; ** = p <0.01; *** = p <0.001; **** = p <0.0001).

OVA-induced allergic airway inflammation was initiated at PND3 as previously described (25, 26) by an *i.p*. injection of 15 μg low endotoxin purified OVA (Worthington; Lakewood, USA) absorbed in 1 mg of Aluminum Hydroxyde adjuvant (ALUM) (Thermo Fisher; Massachusetts, USA). An *i.p.* boost injection of OVA-ALUM or PBS as a control was done on PND17 followed by 3 subsequent intranasal (*i.n.*) challenges with 30 μg OVA (PND27-30) (**Figure 1A**). Allergic reaction features were determined 24h after the last challenge. HDM‐induced asthma was established by *i.n.* sensitization with 10 μg HDM *Dermatophagoides pteronyssinus* extract (Greer Laboratories; Lenoir, USA) on PND3 as previously described (25). This sensitization was followed by 5 consecutive *i.n.* challenges with 50 μg HDM (PND10-14). Allergic airway features were analyzed 72h later.

### Airway hyperresponsiveness determination

Two weeks after the last OVA challenge, mice were challenged again for 2 consecutive days with 30 µg OVA as the mice did not weight enough for a reliable airway hyperresponsiveness (AHR) determination at PND30. AHR to increasing concentrations of nebulized methacholine (0–200 mg/mL) was measured by FlexiVent^©^ apparatus (SCIREQ; Montréal, Canada) 24h later.

### Culture and cytokine secretion measurement

Cell cultures were performed in RPMI-1640 medium containing 10% (vol/vol) FCS, 2 mM glutamine, 1 mM sodium pyruvate, 0.1 mM non-essential amino acids and 40 mM b-mercaptoethanol (Lonza; Basel, Switzerland). Cultures were restimulated with 100 µg/mL OVA grade V (Sigma-Aldrich; Missouri, USA) or 15 μg/mL HDM and kept for 3 days at 37°C in a 5% CO2 atmosphere. IL-5, IL-13 and IFN-γ Duoset ELISA kits (R&D System; Minneapolis, USA) were used to measure respective cytokine secretion in culture supernatant according to the manufacturer’s instructions. The detection range is 2000 pg/ml to 31.3 pg/ml for IL-5 and 4000 pg/ml to 62.5 pg/ml for IL-13 and IFN-γ.The inter and intra CV is ≤15%.

### Real-time quantitative PCR from frozen tissues

Spleens and lungs were harvested and frozen at −80°C. RNA was extracted using automated MagNA Pure LC (RNA Isolation kit III) following the manufacturer’s protocol (Roche; Machelen, Belgium). Reverse transcription and qPCR were performed in a single step using the TaqMan RNA Amplification on Lightcycler^®^ 480 apparatus (Roche). For individual samples, mRNA levels were normalized to those of β-actin and Hypoxanthin-Guanin-Phosphoribosyltransferase (HPRT) used as housekeeping genes. The list of our own designed primers and probes can be found in the **Supplementary Table 1**. For individual samples, relative RNA levels (2^–ΔΔCt^) were determined by comparing a) the cycle thresholds (Ct) for the gene of interest and calibrator gene (ΔCt), the geomean of HPRT and b-actin (for cytokines) or RPL32 (for bacterial DNA), and b) ^2–ΔCt^ values for the experimental group vs. the reference sample (2^–ΔCt^ values mean of the non-treated PBS mice).

### Quantification of intestinal commensal bacteria

Colon content was harvested from PND3 mice, and their mothers supplemented or not with *L.rhamnosus*. Microbial DNA was then extracted using “Nucleospin Microbial DNA” (Macherey-Nagel; Dueren, Germany) kit according to the manufacturer’s protocol. DNA samples were quantified using the NanoDrop™ spectrophotometer and stored at -20°C. These samples were used to quantify total intestinal microbiota by RT-qPCR using “Lightcycler 480 SYBR Green I Master” reaction mix (Roche Diagnostics). Sequence of primers and probes are available on request.

### Quantification of immunoglobulins in the serum

Ig levels were determined by sandwich ELISAs using purified rat IgG1 κ directed against ϵ (clone LO-ME-3, SYnAbs) H chains of mouse IgE as capture mAb and purified biotinylated rat IgG1 κ directed against ϵ (clone LO-ME-2, SYnAbs) H chains for IgE as detection mAb with horseradish peroxidase-avidin conjugate (R&D System). Standard curves were generated with purified IgE (clone C38-2; BD Biosciences). For OVA-specific and HDM-specific IgG1 quantification, OVA-coated (10 μg/mL; Sigma-Aldrich) or HDM-coated (20 µg/mL; Greer Laboratories) plates were incubated with serial dilutions of sera and biotinylated mAb to IgG1 (clone LO-MG1-2, SYnAbs) with horseradish peroxidase-avidin conjugate (R&D System). OVA- or HDM-specific IgG1 titers were expressed as relative values to hyperimmune sera used as reference.

### Flow cytometry

For cell identification, spleen and mediastinal lymph nodes were disrupted using a Pyrex Potter tissue homogenizer (VWR; Leuven, Belgium) while lung tissue was harvested in RPMI‐1640 medium supplemented with recombinant Grade I DNAse I (10U/mL, Thermo Fisher) and collagenase A (1 mg/mL, Roche) and dissociated using the GentleMACS (Miltenyi Biotec; Bergisch Gladbach, Germany) lung programs 1 and 2, with incubation at 37°C for 20 min between both steps. Red blood cells were lysed by Ammonium-Chloride-Potassium lysing Buffer for 1 min and cells of interest were stained with antibody cocktails diluted in FACS buffer (PBS/0,5% BSA/2 mM EDTA) at 4° C in the dark for 20 min. Dead cells were excluded by adding fixable viability dye conjugated to iFluor860 maleimide (AAT Bioquest; California, USA). For intranuclear staining, splenocytes and lymph nodes were fixed and permeabilized using the Foxp3-Staining buffer kit (eBiosciences) following the one-step manufacturer protocol. Antibodies used for flow cytometry are summarized in the **Supplementary Table 2**. FACS gating strategies used for the identification of dendritic cells and helper CD4 T cells in the spleen, in the lung or in the MLN are shown on **Figures S2**, **S4**, **S6** and **S7**. cDC1 are defined as CD45^+^F4/80^-^CD64^-^CD11c^+^MHCII^+^CD26^+^XCR1^+^SIRPa^-^ while cDC2 are defined as CD45^+^F4/80^-^CD64^-^CD11c^+^MHCII^+^CD26^+^XCR1^-^SIRPa^+^. Furthermore, in the neonatal spleen, pre-cDC1 are defined as CD8α^-^ cDC1 cells. Samples were acquired on CytoFLEX LX (6 lasers, Beckman Coulter) and analyzed using Flowjo Software (Tree Star, Inc).

### Statistical analysis

Data are expressed as mean ± SEM. Statistical comparison was done using GraphPad Prism 7 (GraphPad Software; San Diego, USA) and statistical test is specified in the legend of each Figure. Shapiro-Wilk test was used for normality test of the data and to choose the appropriate statistical test. *P* values less than or equal to 0.05 were considered significant.

## Results

### *L.rhamnosus* supplementation prevents neonatal type 2 allergic airways reaction to OVA

We daily fed pregnant and breastfeeding mice from late gestational stage (embryonic day 16) to 3 days post-partum with 2.10^8^ CFU of *L.rhamnosus*. Firstly, by measuring copies of *Eubacter* 16S rDNA in colon content from the mothers and their neonates, we have shown that *L.rhamnosus* supplementation did not affect total bacteria (**Figure S1**). We then compared allergic airway reaction severity in neonates from mothers supplemented or not with the *L.rhamnosus*. For this, 3-day-old neonates were *i.p.* sensitized with OVA-ALUM with a recall on day 17 of life and were 3 times *i.n.* OVA challenged from 10 days later (**Figure 1A**). The type 2 immunity hallmarks of allergic airways disease were monitored 24h after the first round of challenges (PND30). Strikingly, when the mothers were supplemented with *L.rhamnosus*, features of allergic airway disease were remarkably decreased in their offspring exposed intranasally with OVA allergens. Indeed, *L.rhamnosus* supplementation inhibited the lung eosinophilia (with no impact on neutrophil and lymphocyte numbers) (**Figure 1B**) and the Th2-associated mRNA transcripts (i.e., *Il4* and *Il5* and a trend for *Il13*) in mice neonatally sensitized with OVA (**Figure 1C**). The maternal *L.rhamnosus* treatment also reduced the total serum IgE and the OVA-specific IgG1 (**Figure 1D**) and decreased the Th2-cell-associated cytokines in mediastinal lymph nodes (MLN) stimulated with OVA (**Figure 1E**) compared with no maternal supplementation. AHR, monitored after a second round of 2 *i.n.* challenges (starting on day 42 of life, **Figure 1A**) by increases in respiratory system resistance and elastance and alveolar tissue damping in response to methacholine, was also markedly lower in neonates from *L.rhamnosus*-treated mothers (**Figure 1F**).

### Maternal *L.rhamnosus* supplementation favored neonatal splenic cDC1 maturation and Th1 differentiation

**Figure 2:**
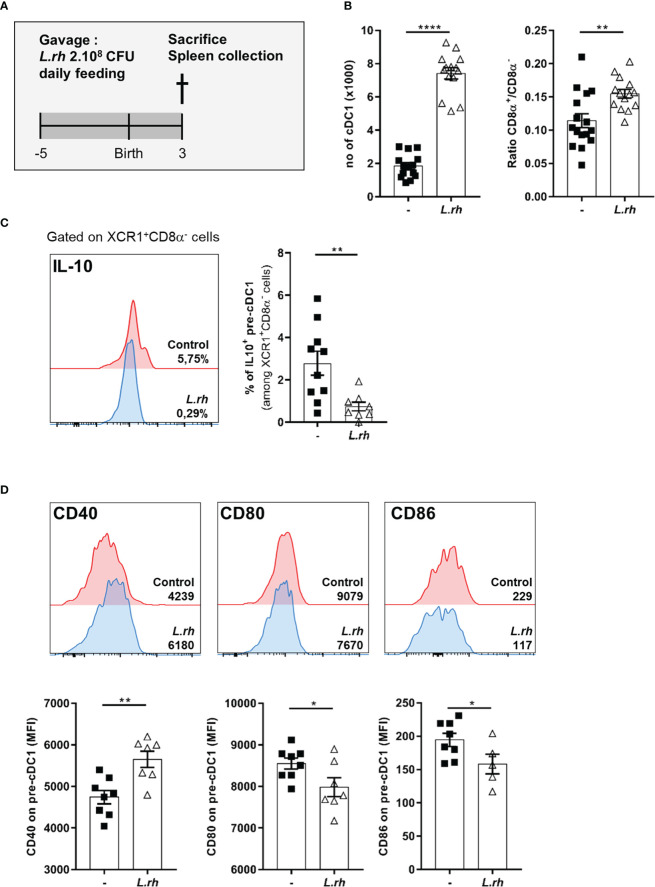
Maternal *L.rhamnosus* supplementation enhanced splenic pre-cDC1 differentiation and functional maturation. **(A)** Pregnant females were orally administered daily with 2.10^8^ CFU of *Lactobacillus rhamnosus* VES001 from 5 days before to 3 days after delivery. The spleens of PND3 neonates born from control (-) or *L.rhamnosus*-treated (*L.rh*) mothers were collected and stained for FACS analysis. **(B)** Total number of splenic cDC1 cells, defined as CD45^+^F4/80^-^CD64^-^CD11c^+^MHCII^+^CD26^+^XCR1^+^SIRPa^-^(CD8^+/-^) cells, and CD8α^+^/CD8α^-^ ratio among neonatal cDC1. **(C)** Representative single histograms and percentages of IL-10 producing pre-cDC1 were determined in the spleen of IL-10^reporter/GFP^ PND3 neonates. The percentages of IL-10 positive cells among pre-cDC1 of the representative samples are shown in the single histograms. **(D)** Representative single histograms and MFI of CD40, CD80 and CD86 co-stimulation markers in pre-cDC1 gated population. The MFI of representative samples are shown on the single histograms. Results are a pool of 2–3 independent experiments and are shown as mean ± SEM. Statistical analysis was done using an unpaired t test or a Mann-Whitney test. (* = p<0.05; ** = p<0.01; **** = p<0.0001).

We hypothesized that maternal supplementation with *L.rhamnosus* could represent a stimulus to educate neonatal cDC compartment (**Figure 2A**). We observed that *L.rhamnosus* increased the number of splenic cDC1 in PND3 neonates (**Figure 2B**) as well as their differentiation and functional maturation. We previously shown in mice that cDC1 do not express CD8α (**Figure S2**, called pre-cDC1) and produce IL-10 during the first week of life (5). Here, we observed that the *L.rhamnosus* treatment enhanced the CD8α^+^/CD8α^-^ ratio (**Figure 2B**) and increased the expression of CD40 (but not CD80 and CD86) among these pre-cDC1 (**Figure 2D**). This phenomenon is correlated with a cDC1 differentiation and is further reinforced by a reduced proportion of IL-10 producing pre-cDC1 (**Figure 2C**). Despite their 10 times lower frequency in the spleen, cDC2 number was also increased by *L.rhamnosus* with an increase of costimulatory molecules such as CD40, CD80 and CD86 (**Figure S3**).

**Figure 3:**
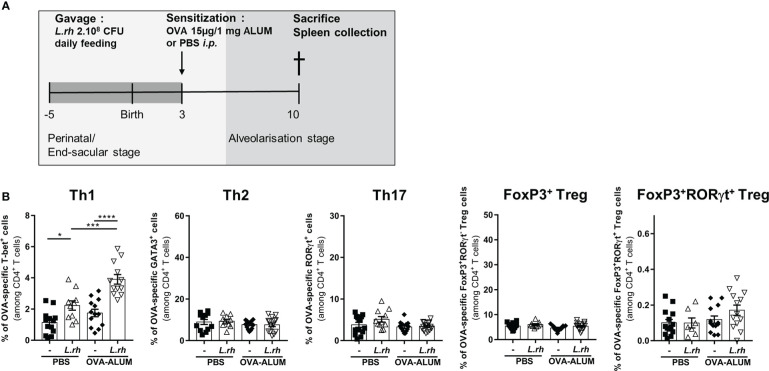
Maternal *L.rhamnosus* treatment promotes a Th1 polarization in the neonatal spleen. **(A)** Neonates born from control (-) or probiotic-supplemented mother (*L.rh*) were sensitized on PND3 with 15µg OVA in 1mg ALUM and sacrificed 1 week later. **(B)** Percentages of OVA-specific CD4^+^ T cells expressing the transcription factors associated to Th1 (T-bet), Th2 (GATA3), Th17 (RorγT), conventional Treg (FoxP3) and RORγt Treg (FoxP3 and RORγt) were determined by FACS in the spleen on PND10. Results are a pool of 4 independent experiments and are shown as mean ± SEM. Statistical analysis was done using an ordinary one-way ANOVA test (Holm-Sidak’s multiple comparisons test) or a Kruskal-Wallis test (Dunn’s multiple comparisons test) (* = p<0.05; *** = p<0.001;, **** = p <0.0001).

We further investigated the influence of *L.rhamnosus* on the CD4^+^ T-cell polarization in early life based on their master transcriptional regulators expression (**Figure S4**). For this, neonates from *L.rhamnosus*-supplemented or control mothers were sensitized at PND3 with OVA-ALUM and the polarization of OVA-specific CD4^+^ T-cell subsets was evaluated 1 week later in the spleen (**Figure 3A**). In OVA-vaccinated newborns, maternal *L.rhamnosus* supplementation had no impact on GATA-3^+^ Th2, RORγt^+^ Th17 or Foxp3^+^ Treg cells frequencies but it resulted in the increased frequency of OVA-specific T-bet^+^ Th1 cells in link with the increased maturation of splenic pre-cDC1 (**Figure 3B**). We noticed a slight but not significant increase of RORγt^+^Foxp3^+^ Treg cells frequency compared with no supplementation (**Figure 3B**). Interestingly, that specific pro-Th1 effect of maternal *L.rhamnosus* treatment was maintained in the spleen of the offspring until adulthood as witnessed by an increased IFN-γ secretion and a decreased IL-5 and IL-13 production in response to OVA of PND30 immunized mice compared with no supplementation (**Figure S5**).

Together, these results indicate that perinatal exposure to *L.rhamnosus* drives the functional maturation of neonatal splenic pre-cDC1 and the polarization of Th1 subset of CD4^+^ T-cells. The *L.rhamnosus* treatment also increases the amount of splenic cDC2 and shows a tendency to increase Foxp3^+^RORγt^+^ CD4^+^ T cells.

### Maternal *L.rhamnosus* supplementation impacts neonatal pulmonary cDC compartments and increases FoxP3^+^ RORgt^+^ Treg

**Figure 4:**
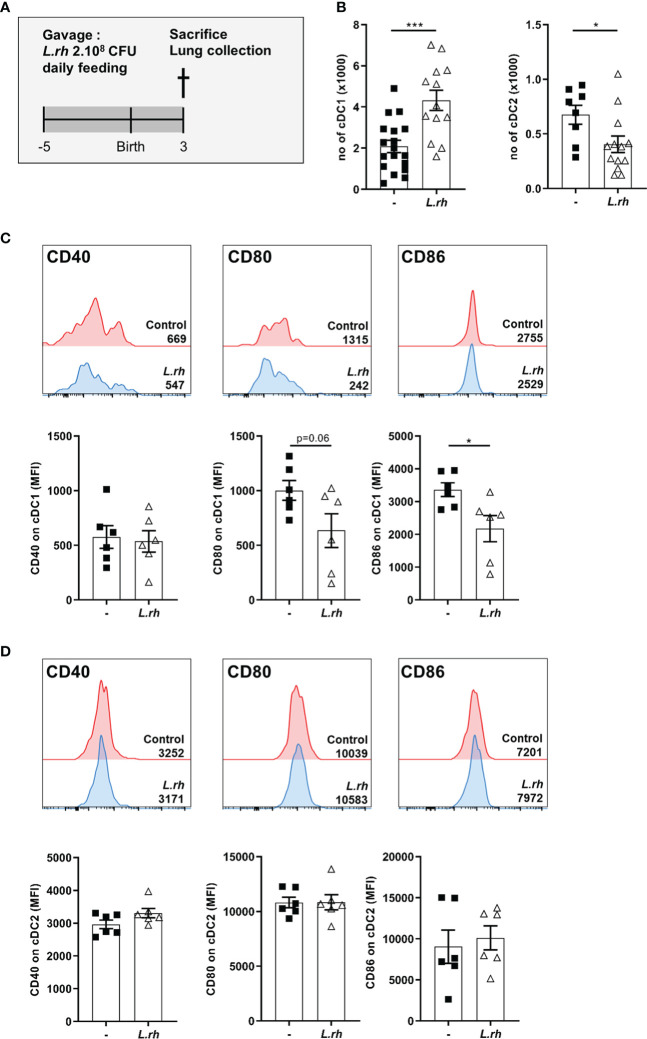
Influence of maternal *L.rhamnosus* supplementation on dendritic cell subsets in the neonatal lung. **(A)** Pregnant females were orally administered daily with 2.10^8^ CFU of *Lactobacillus rhamnosus* VES001 from 5 days before to 3 days after delivery. The lungs of PND3 neonates born from control (-) or *L.rhamnosus*-treated (*L.rh*) mothers were collected, digested and stained for FACS analysis. **(B)** Total number of cDC1 and cDC2, defined as CD45^+^F4/80^-^CD64^-^CD11c^+^MHCII^+^CD26^+^XCR1^+^SIRPa^-^ and CD45^+^F4/80^-^CD64^-^CD11c^+^MHCII^+^CD26^+^XCR1^-^SIRPa^+^ cells respectively. **(C, D)** Representative single histograms and MFI of CD40, CD80 and CD86 markers in cDC1 **(C)** and cDC2 **(D)** gated population. The MFI of representative samples are shown on the single histograms. Results are a pool of 2–3 independent experiments and are shown as mean ± SEM. Statistical analysis was done using an unpaired t test or a Mann-Whitney test. (* = p<0.05; *** = p<0.001).

Next, we explored the impact of maternal *L.rhamnosus* supplementation on the offspring mucosal immunity. First, we observed that the *L.rhamnosus* treatment increased the number of pulmonary cDC1 in PND3 neonates (**Figures 4A, B**; **Figure S6**) but without impact on their CD40 expression and with downregulation of their CD80/86 expression (**Figure 4C**). Moreover, the number of pulmonary cDC2, already low compared to cDC1, was drastically decreased in neonates from *L.rhamnosus* treated mothers (**Figure 4A**) while no impact on their co-stimulation markers expression was observed (**Figure 4D**).

**Figure 5:**
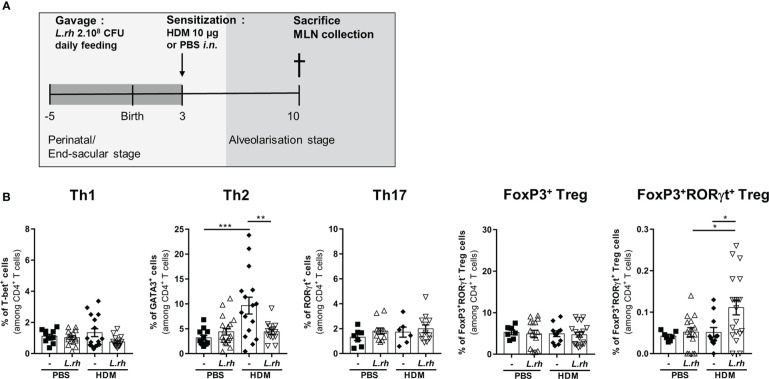
Maternal *L.rhamnosus* supplementation inhibits T cell polarization in the neonatal lung. **(A)** Neonates born from control **(-)** or *L.rhamnosus*-supplemented (*L.rh*) mothers were sensitized on PND3 with 10 µg HDM and sacrificed 1 week later. **(B)** Percentages of CD4^+^ T cells expressing the transcription factors associated with Th1 (T-bet), Th2 (GATA3), Th17 (RorγT), conventional Treg (FoxP3) and RORγt Treg (FoxP3 and RORγt) cells were determined by FACS in the MLN of PND10 neonates. Results are a pool of 2–4 independent experiments and are shown as mean ± SEM. Statistical analysis was done using an ordinary one-way ANOVA test (Holm-Sidak’s multiple comparisons test) or a Kruskal-Wallis test (Dunn’s multiple comparisons test) (* = p <0.05; ** = p <0.01; *** = p <0.001).

To determine the impact of maternal *L.rhamnosus* exposure on neonatal CD4^+^ T-cell polarization (**Figure S7**), PND3 mice from control or *L.rhamnosus* treated mothers were *i.n.* sensitized to house dust mite (HDM) extracts and their MLN were collected 7 days later (**Figure 5A**). We observed an inhibition of the GATA-3^+^ Th2 responses upon HDM stimulation in HDM-sensitized neonates from *L.rhamnosus* treated mothers compared to neonates from untreated mothers (**Figure 5B**). The *L.rhamnosus* treatment had no impact on the CD4^+^ T-cell frequencies of T-bet^+^ Th1, RORγt^+^ Th17 or Foxp3^+^ Treg. In contrast, RORγt^+^Foxp3^+^ CD4^+^ T cells were significantly induced in HDM-sensitized neonates upon maternal *L.rhamnosus* supplementation (**Figure 5B**).

Together, these results indicate that perinatal exposure to *L.rhamnosus* limits pulmonary cDC1 maturation, decreases the cDC2 subset with the expansion of a RORγt^+^Foxp3^+^ Treg subset conducting to the inability to activate a Th2-type response to inhaled HDM.

### Maternal *L.rhamnosus* supplementation prevents neonatal type 2 allergic airways reaction to HDM allergens

**Figure 6:**
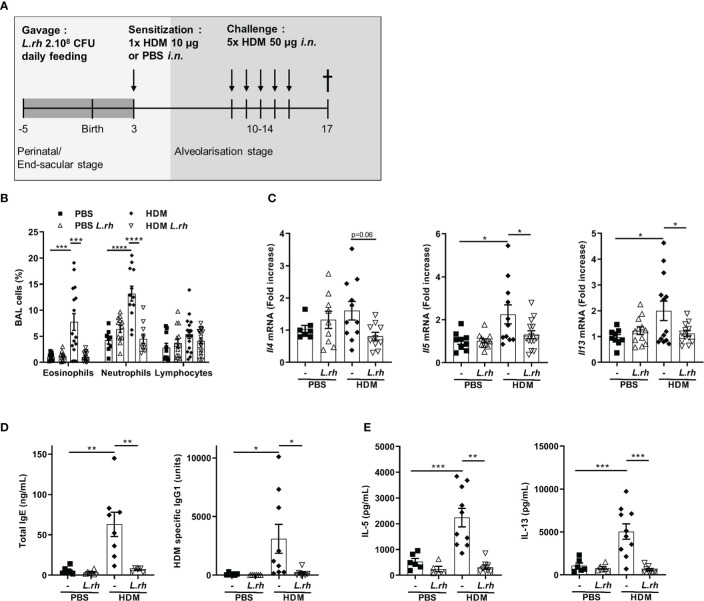
Perinatal *L.rhamnosus* exposures prevents neonates from type 2 responses to HDM. **(A)** Protocol of allergic asthma induction. Neonates born from C57BL/6 control (-) or probiotic-supplemented mother (*L.rh*) were mock-sensitized with PBS or sensitized with 10 µg HDM on PND3 and were challenged with 50 µg HDM on 5 consecutive days one week after sensitization. Mice were sacrificed on PND17. **(B)** Cell percentages in the BAL were determined 72h after the last challenge by FACS. **(C)** Pulmonary mRNA normalized expression of *Il4*, *Il5* and *Il13* were analyzed by qRT-PCR. Relative units were obtained by comparing each group to the mean of non-treated PBS mice. **(D)** Total IgE and HDM-specific IgG1 in serum were obtained by ELISA. **(E)** Th2-associated cytokines in MLN restimulated for 3 days with 15 µg/mL HDM were measured by ELISA. Results are a pool of 3–4 independent experiments and are shown as mean ± SEM. Statistical analysis was done using an ordinary one-way ANOVA (Holm-Sidak’s multiple comparisons test) or a Kruskal-Wallis test (Dunn’s multiple comparisons test) (* = p <0.05; ** = p <0.01; *** = p <0.001; **** = p <0.0001).

Finally, to evaluate the beneficial effects of maternal *L.rhamnosus* supplementation on the allergic airways disease in the offspring, we used the HDM model which replicate many of the features of asthma: increased number of eosinophils and neutrophils in bronchoalveolar lavage fluid, increased Th2 cytokines levels in the lung and from mediastinal lymph node (MLN) T cells and increased IgE levels. For that, PND3 mice from control or *L.rhamnosus*-treated mothers were *i.n.* sensitized with HDM extracts, which contains the most common triggers of allergic asthma in humans. One week later, they were *i.n.* challenged with HDM on 5 consecutive days and 3 days later, allergic airway inflammation was evaluated (**Figure 6A**). Interestingly, *L.rhamnosus* maternal supplementation resulted in decreased eosinophilia and neutrophilia in the bronchoalveolar lavage (BAL) fluid (**Figure 6B**) and in decreased Th2-associated cytokines mRNA transcripts (i.e., *Il4*, *Il5* and *Il13*) in the lung of neonatally sensitized mice (**Figure 6C**). Moreover, *L.rhamnosus* also induced a reduction in total IgE and HDM-specific IgG1 (**Figure 6D**) and in secretion of Th2-cell-associated cytokines in MLN (**Figure 6E**) compared with HDM-sensitized mice from non-treated mothers. Therefore, we confirmed that maternal *L.rhamnosus* supplementation also decreased type 2 immunity to a physiologically relevant allergen when delivered locally.

## Discussion

“Hygiene hypothesis”, a concept highlighting the importance of microbial exposure in the development of an appropriate immune response in early life, could account for the increased prevalence of atopic asthma worldwide (12, 27, 28). Therefore, the use of probiotics, defined as “live microorganisms which when administered in adequate amounts confer a health benefit on the host” (29), has been suggested to reduce the risk of allergies development in early life. One of the most studied probiotic strains for its potential immunomodulatory function is *L.rhamnosus*. Despite the effects on allergic airways disease have not yet been demonstrated in humans (22, 23, 30, 31), studies conducted in adult mice have shown a protective effect of *L.rhamnosus* on AHR in link with a decreased airway inflammation and Th2 cytokines (32–35). No studies showed the impact of solely maternal *L.rhamnosus* supplementation on the development of asthma in offspring when initiated early in life. Indeed, a previous study has shown that perinatal maternal *L.rhamnosus* supplementation suppresses allergic airway inflammation in young adult offspring while Th2 bias responses to allergens as well as AHR remained unchanged (24). However, in their study design, the sensitization occurs after weaning, at 25 days of life. Here, we found that maternal *L.rhamnosus* supplementation during gestation and breastfeeding prevents type 2 immunity and allergy airway inflammation to systemically administered and inhaled allergens early in life. In the case of OVA allergens, perinatal *L.rhamnosus* exposure prevents AHR development. This allergic airway reaction prevention involves a functional maturation of neonatal splenic pre-cDC1, inhibiting IL-10 production and inducing a preferential Th1 polarization that prevents type 2 immunity to OVA. On the other hand, the prevention of HDM-induced asthma occurs by altering the pulmonary cDC maturation and through the induction of RORγt^+^FoxP3^+^ Treg cells.

Among all cell types implicated in the initiation and maintenance of type 2 allergic disorders, cDC represent the most crucial drivers for the induction of allergic Th2 cell response to allergens (36). Our study is the first demonstration that maternal *L.rhamnosus* supplementation impacts neonatal cDC maturation in the spleen and in the lung. It is well established that pulmonary cDC2 are necessary and sufficient to induce allergic sensitization (37). On the other side, cDC1, through IL-12 production and the resulting Th1 responses, attenuate allergic airway inflammation by inhibiting aberrant Th2 immunity (38). However, during the first week of life, IL-12 production is delayed and restrained by IL-10 secretion leading to an intrinsic bias to generate Th2 responses (3, 5) and a propensity to develop asthma. Upon maternal *L.rhamnosus* supplementation, we showed that the neonatal cDC1 compartment is increased and more differentiated as shown by the upregulated CD8α expression while IL-10 producing pre-cDC1 are decreased in the spleen. Furthermore, neonatal pre-cDC1 exhibit higher levels of CD40 on their surface but downregulate CD80/CD86 co-stimulatory markers. To the best of our knowledge, this is the first time that the maturation of splenic neonatal dendritic cells is observed *in vivo* upon oral *L.rhamnosus* maternal supplementation. One potential mechanism could be trough the induction of mibrobiota-derived maturating cytokines such as TNF-α (39). It reinforces data collected *in vitro* from both murine bone-marrow derived DC and human monocyte-derived DC stimulation with Lactobacilli that induced the upregulation of CD40, CD80 and CD86 (40, 41). Although very underrepresented, the increased frequency of splenic cDC2 observed in neonates after *L.rhamnosus* maternal supplementation could also be attributed to microbiota-derived cytokines such as TNF-α. It has been shown that CD80/CD86 expression on cDC is required for the sensitization to OVA by playing an essential role in the induction of Th2 differentiation of naive OVA-specific T cells (42). On the other hand, CD40 signaling, expressed mainly by activated cDC, is involved in Th1 differentiation (43) and in the suppression of allergic airway inflammation (44). This is in line with our observation as naive OVA-specific T cells preferentially differentiate into Th1 cells in the spleen upon maternal *L.rhamnosus* treatment. This early life Th1 polarization is sufficient to counterbalance allergens specific Th2 response and airway inflammation later in life. Finally, we strengthen our previous study by demonstrating the importance of microbiota colonization for neonatal splenic pre-cDC1 functional maturation (39). The positive effects of maternal *L.rhamnosus* treatment in the offspring are caused directly by bacterial components or indirectly by mediators produced in the mother and transferred to the child. Several studies have shown that since 13 weeks of human gestation and in the immediate postnatal period, the translocation of intestinal microorganisms, as well as their derived metabolites, is increased and can thus be transferred to the unborn fetus *via* the placenta (45) or to the newborn mice through maternal milk (46). Concerning the impact of metabolites, to date, only one study has demonstrated the interplay between maternally derived acetate and protection against asthma in neonates. Mechanistically, acetate suppresses allergic airway disease in adult offspring by enhancing the Treg cells number and function. This beneficial effect was mediated *in utero* as supplementation during lactation only had no effect (21). Besides, maternal probiotics supplementation can also increase the bioavailability of micronutrients such as vitamin B12, folate, calcium, iron, and zinc which can have a positive effect in the prevention of Th2-mediated disease in offspring (47–49). However, it remains to be investigated how *L.rhamnosus* impacts the bioavailability of nutrients.

Finally, we confirmed that maternal *L.rhamnosus* supplementation has a beneficial effect on the development of asthma with a physiologically relevant inhaled allergen as we observed a protective effect of *L.rhamnosus* on type 2 responses and airway inflammation in a murine HDM asthma model. However, the mechanisms involved in the prevention are quite different and implies lung immunity rather than systemic immunity as allergen is delivered locally. In the neonatal lung, cDC2 as well as ILC2 are the main immunological actors in the development of allergic Th2 responses (8). At PND3, maternal *L.rhamnosus* supplementation decreases the number of cDC2 in the lung. In parallel, cDC1 are increased but not functional as the level of co-stimulatory CD80/CD86 markers is decreased and CD40 remained unchanged. Thus, the decreased number of cDC2 combined with the inability of cDC1 to promote T cell polarization led to none Th1 or Th2 allergen-specific T-cell differentiation. However, the probiotic was responsible for a significant induction of RORγt^+^Foxp3^+^ Treg within the MLN. These RORγt^+^ Treg were initially described in the draining lymph nodes from the intestine (50, 51). This Treg subset is negatively regulated by Th1 cells (42) but it remains elusive which cDC regulate its differentiation. In the intestine, Runx/Cbfβ functions in cDC were demonstrated to be essential for the differentiation of intestinal CD103^+^CD11b^+^ cDC2 and for the priming of RORγt-expressing T cells (52) but it has not yet been explored in the lung. Moreover, RORγt^+^Foxp3^+^ Treg were demonstrated to downregulate CD80/86 on cDC (11). This could be a mechanism explaining how RORγt^+^ Treg differentiation may exclude Th1 and Th2 differentiation (11, 51). Interestingly, the induction of RORγt^+^ Treg were observed in the intestine during the weaning when the density and diversity of the colonic microbiota change significantly with the uptake of solid food. During that event, a transient microbiota-mediated innate immune stimulation with enhanced expression of TNF-α and IFN-γ, the activation of retinaldehyde dehydrogenases (RALDH)-positive CD103^+^ cDC1 and cDC2 able to convert vitamin A and increasing levels of microbiota-derived short chain fatty acids (SCFAs), all favor the appearance of RORγt^+^ Treg cells. This age-restricted process of neonatal Treg-cell induction was demonstrated to confer protection from immune-mediated diseases in adult life (53). Therefore, it suggests that perinatal *L.rhamnosus* exposure mimic the weaning reaction and may prevent immune disorder such as airway allergic inflammation through the induction of RORγt^+^ Treg cells.

Taken together, our findings suggest that maternal probiotic supplementation has the potential to modulate in their very young offspring the pulmonary cDC maturation to promote type 3 response to inhaled allergens or to favor type 1 immune response to OVA allergens in the spleen. This neonatal immune maturation through microbiota modulation might represent a new preventive approach to the development of immune-mediated diseases later in life. Importantly, it highlights the fact that the influence of commensals on immune cells is tissue specific, which must be considered when developing probiotic-based interventions for early life.

## Data availability statement

The raw data supporting the conclusions of this article will be made available by the authors, without undue reservation.

## Ethics statement

The animal study was reviewed and approved by Biopole ULB Charleroi Institutional Animal Care and Local Use Committee.

## Author contributions

JS and VF contribute to the concept and the design of the research. JS, CV, SD, AK, and JP performed experiments and procedures. JS, AK, and VF contribute to the writing of the manuscript. All authors have approved the final version of this manuscript.
