# Supplementary figures and images for: Maternal Lactobacillus rhamnosus administration impacts neonatal CD4 T-cell activation and prevents murine T helper 2-type allergic airways disease
Source: Front Immunol. 2023 Jan 4;13:1082648. doi: 10.3389/fimmu.2022.1082648 (PMC9847498; doi:10.3389/fimmu.2022.1082648)

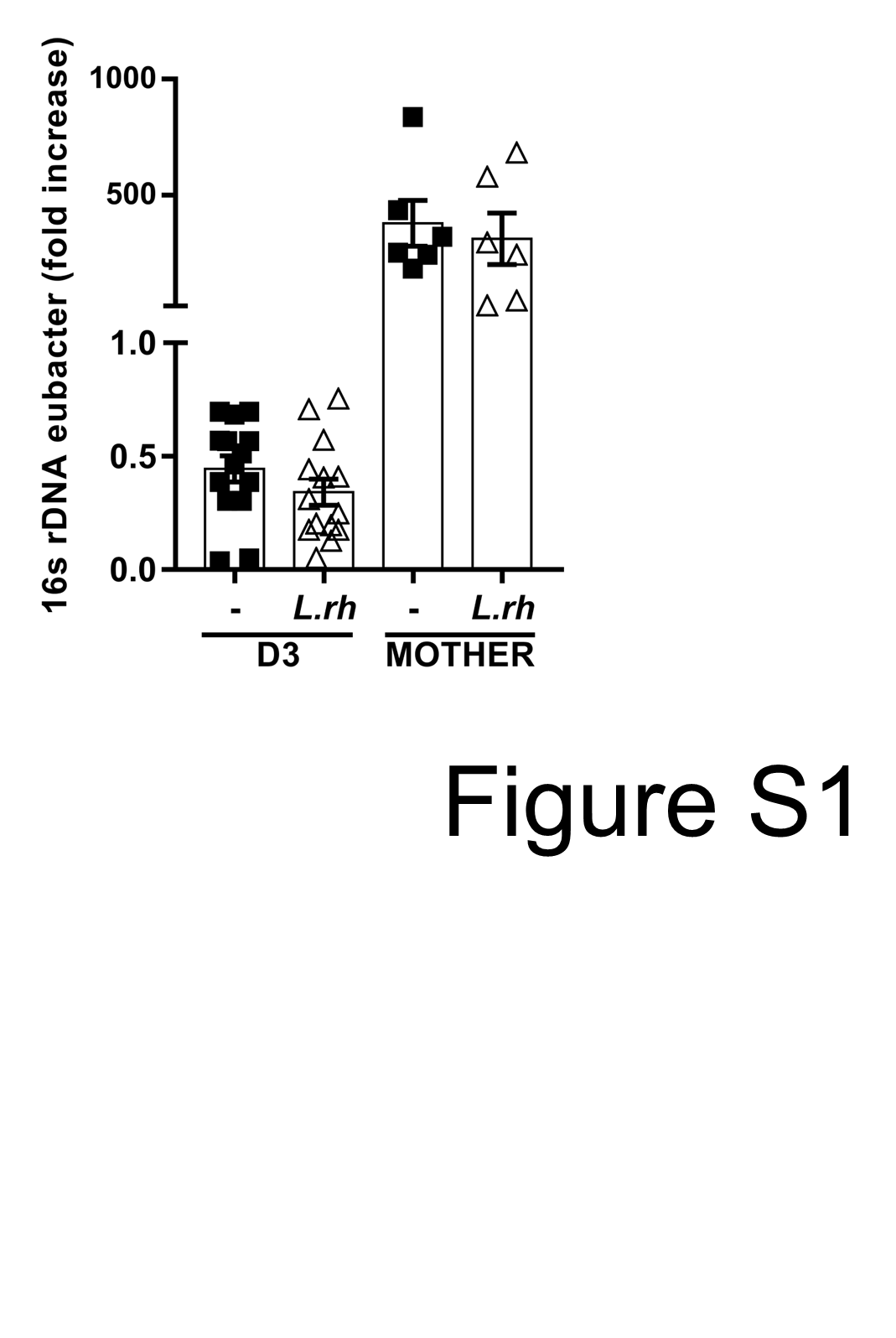

Supplement: Supplementary file 1 [file Image_1.tif]

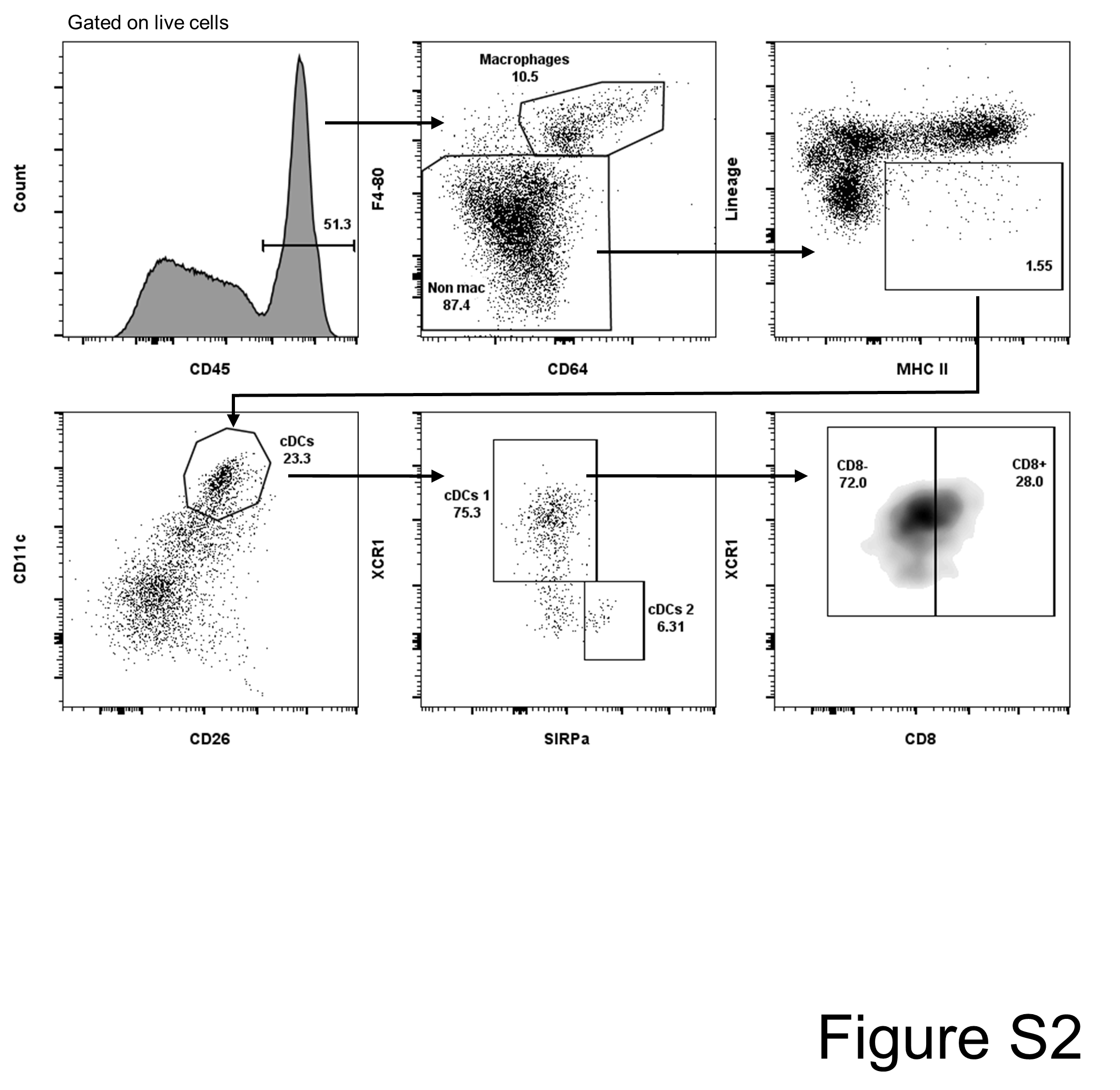

Supplement: Supplementary file 2 [file Image_2.tif]

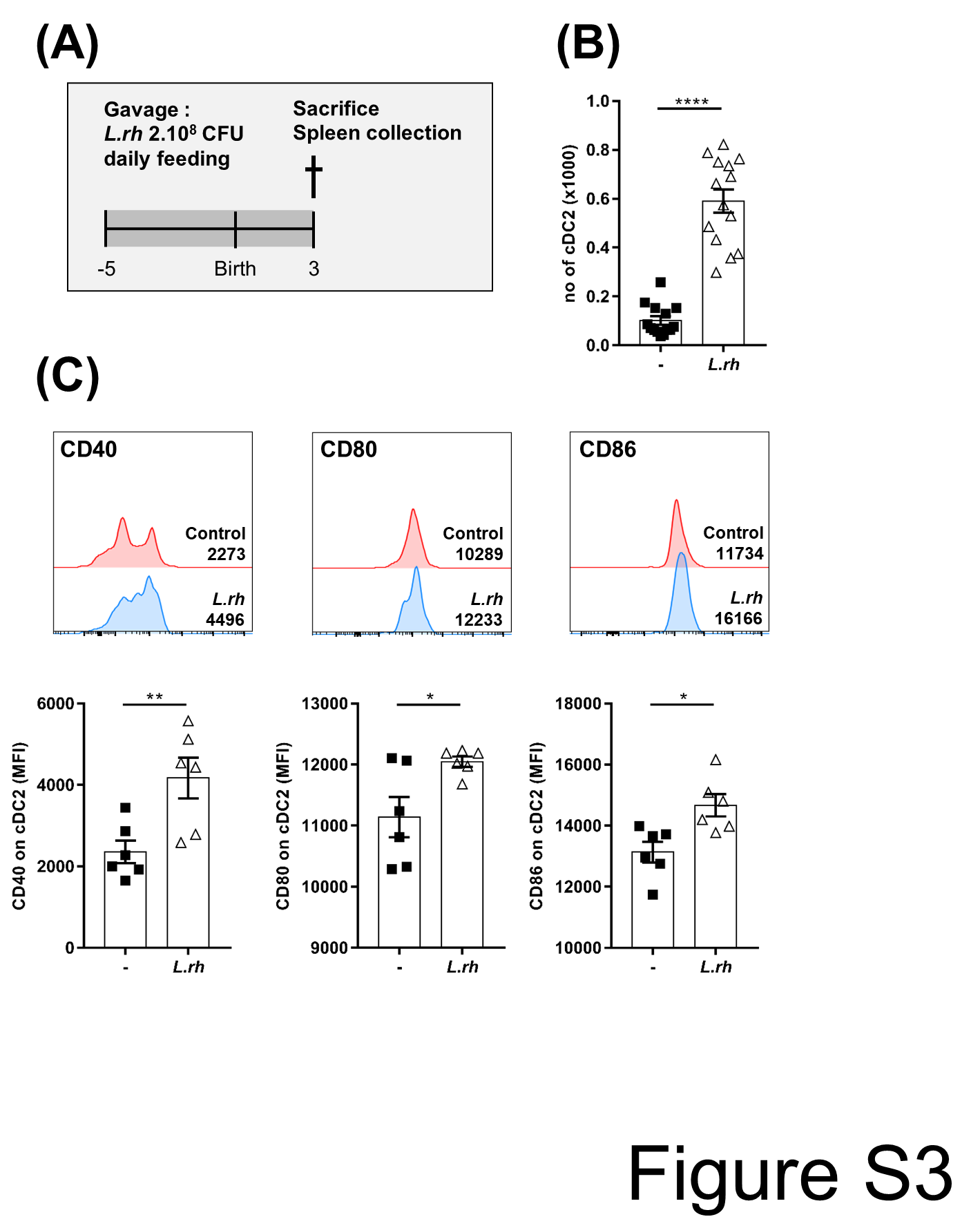

Supplement: Supplementary file 3 [file Image_3.tif]

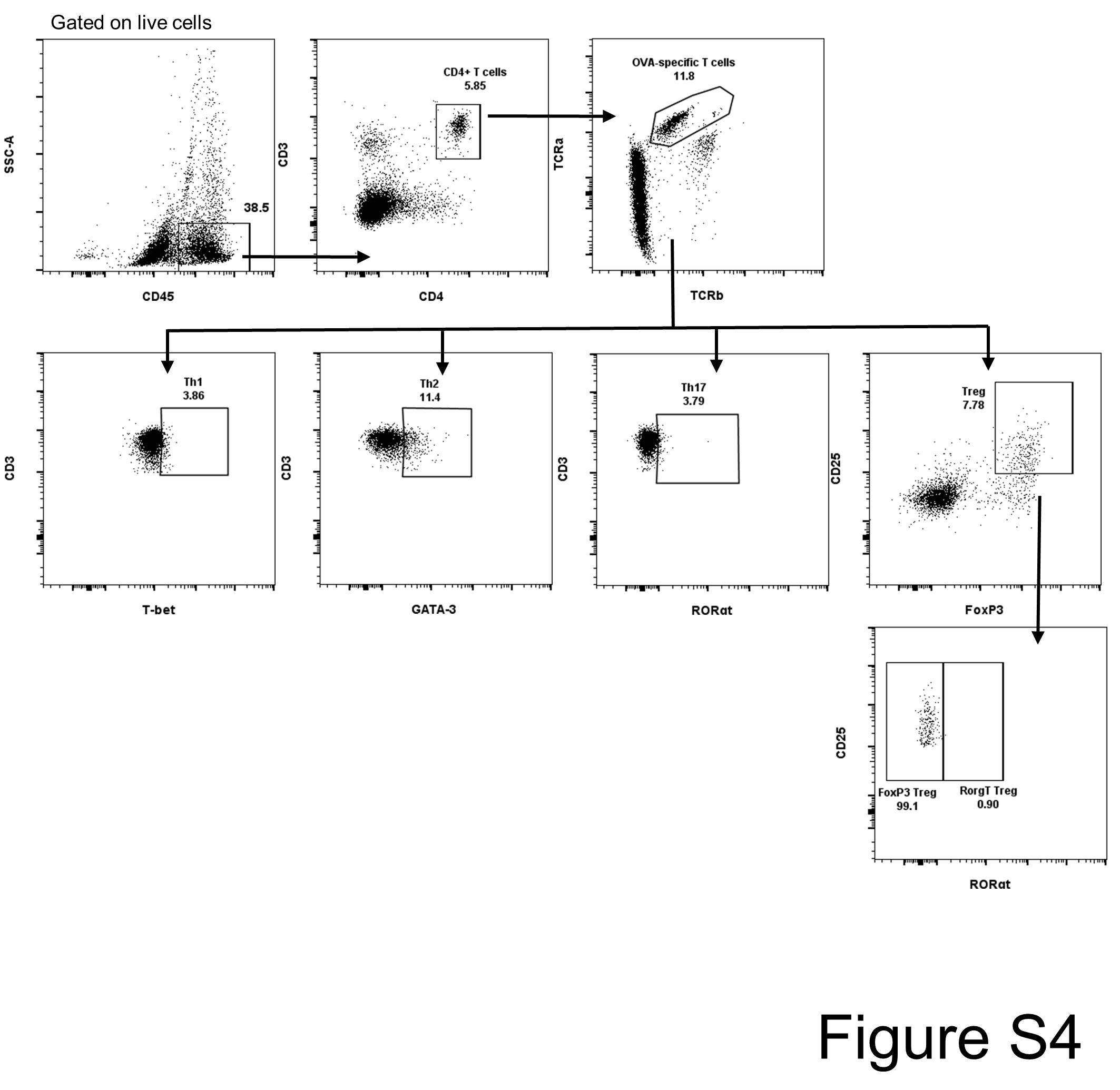

Supplement: Supplementary file 4 [file Image_4.tif]

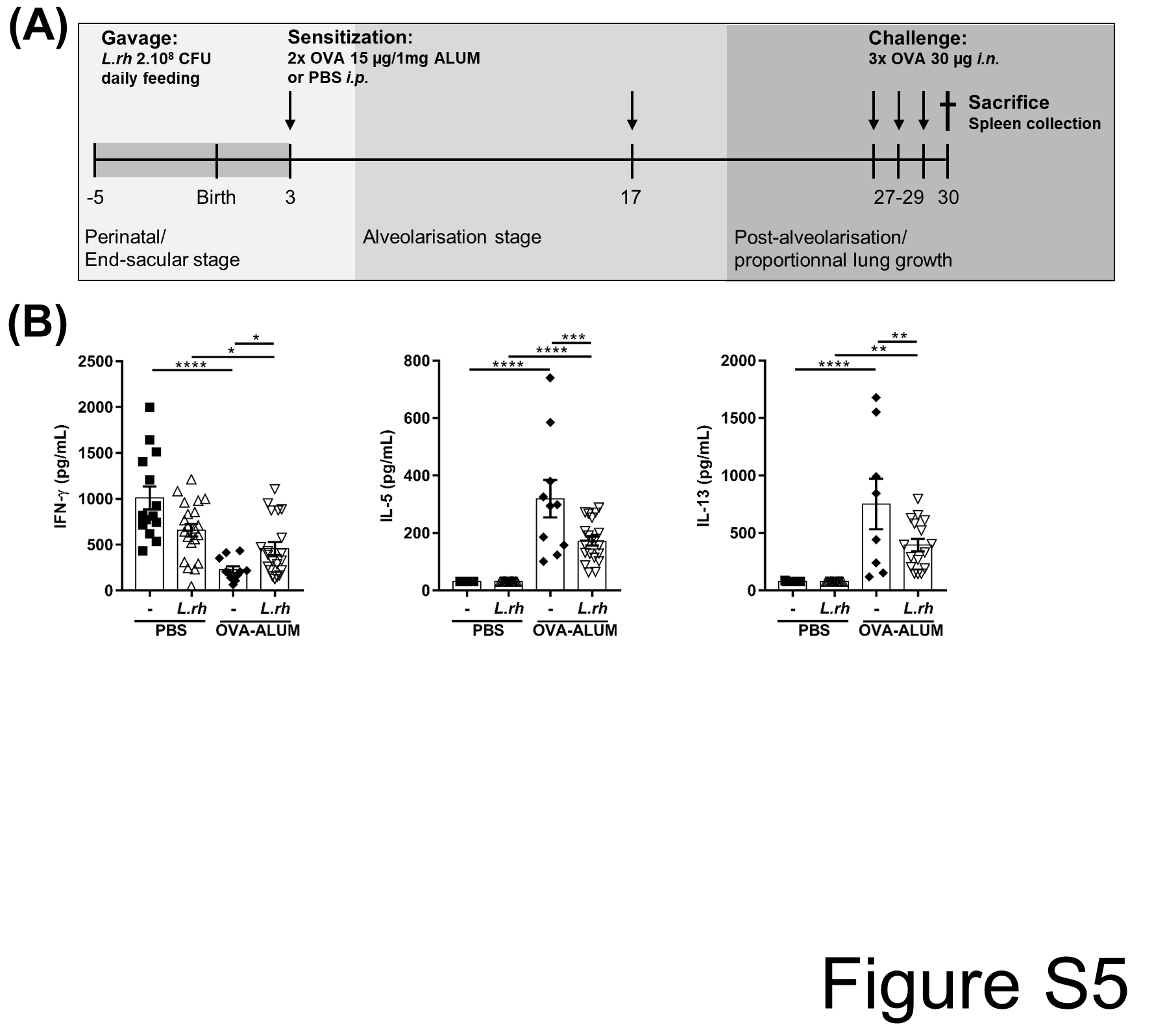

Supplement: Supplementary file 5 [file Image_5.tif]

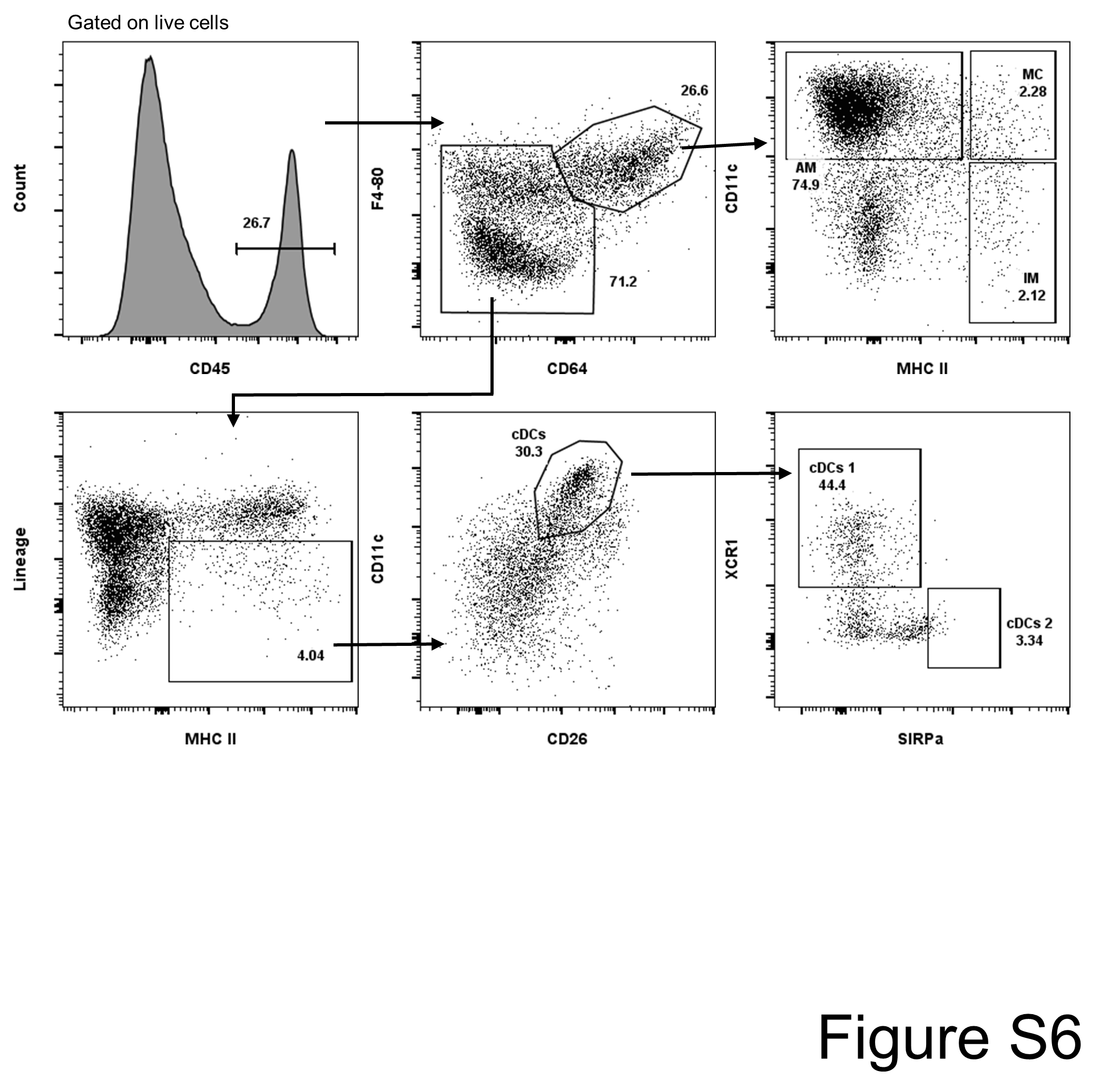

Supplement: Supplementary file 6 [file Image_6.tif]

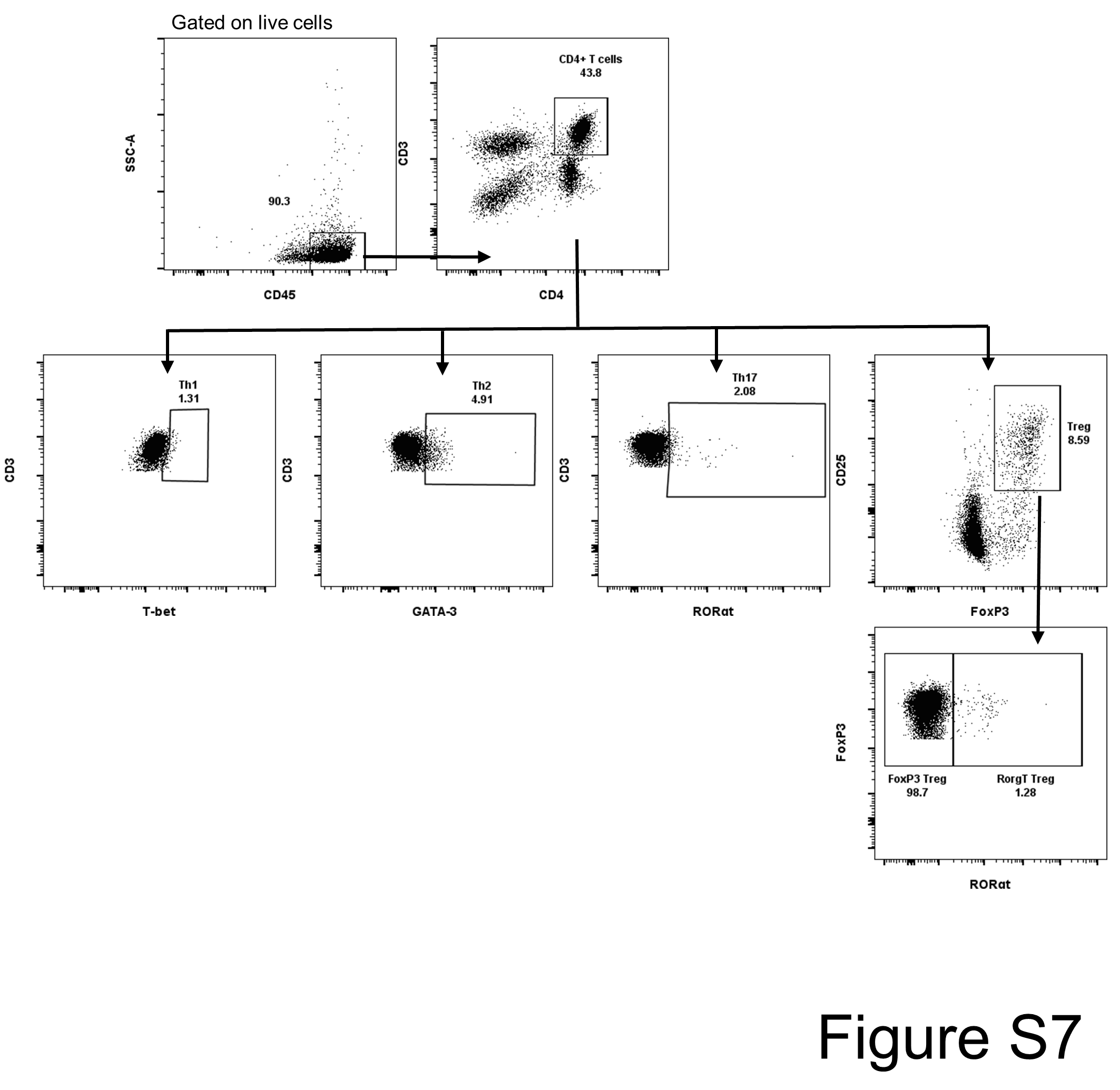

Supplement: Supplementary file 7 [file Image_7.tif]
