# Supplementary material for: Maternal Lactobacillus rhamnosus administration impacts neonatal CD4 T-cell activation and prevents murine T helper 2-type allergic airways disease
Source: Front Immunol. 2023 Jan 4;13:1082648. doi: 10.3389/fimmu.2022.1082648 (PMC9847498; doi:10.3389/fimmu.2022.1082648)
Supplement: Supplementary file 8 [file Table_1.docx]

| **Gene** | **Forward** | **Reverse** | **Probe** |
| --- | --- | --- | --- |
| IL4 | AGA-TCA-TCG-GCA-TTT-TGA-ACG | TTT-GGC-ACA-TCC--ATC-TCC-G | TCA-CAG-GAG-AAG-GGA-CGC-CAT-GC |
| IL5 | GAG-CTC-TGT-TGA-CAA-GCA-ATG | CCA-ATG-CAT-AGC-TGG-TGA-TTT | CGA-TGA-GGC-TTC-CTG-TCC-CTA |
| IL13 | GAG-CTG-AGC-AAC-ATC-ACA-CAA | GCC-AGG-TCC-ACA-CTC-CAT-AC | CCC-TGT-GCA-ACG-GCA-GCA-TG |
| HPRT | GGA-CCT-CTC-GAA-GTG-TTG-GAT | CCA-ACA-ACA-AAC-TTG-TCT-GGA-A | CAG-GCC-AGA-CTT-TGT-TGG-ATT-TGA-A |
| Actine | TCC-TGA-GCG-CAA-GTA-CTC-TGT | CTG-ATC-CAC-ATC-TGC-TGG-AAG | ATC-GGT-GGC-TCC-ATC-CTG-GC |
| RPL32 | GGC-ACC-AGT-CAG-ACC-GAT-AT | CAG-GAT-CTG-GCC-CTT-GAA-C |  |
| Eubacter | ACT-CCT-ACG-GGA-GGC-AGC-AGT | ATT-ACC-GCG-GCT-GCT-GGC |  |

**Supplementary table 1:** List of the sequences of primers and probes.

| **Marker** | **Fluorochrome** | **Clone** | **Company** | **Dilution** |
| --- | --- | --- | --- | --- |
| B220 | BV650 | RA3-6B2 | BD Horizon™ | 1/200 |
| B220 | FITC | RA3-6B2 | BD Pharmingen™ | 1/500 |
| CD11b | Alexa Fluor® 700 | M1/70 | BD Pharmingen™ | 1/500 |
| CD11b | PE-CF594 | M1/79 | BD Horizon™ | 1/500 |
| CD11c | BV421 | N418 | BD Horizon™ | 1/100 |
| CD172a | BV480 | P84 | BD OptiBuild™ | 1/500 |
| CD19 | BV711 | 1D3 | BD Horizon™ | 1/500 |
| CD19 | FITC | 1D3 | BD Pharmingen™ | 1/500 |
| CD25 | BB515 | PC61 | BD Horizon™ | 1/500 |
| CD26 | BV786 | H194-112 | BD OptiBuild™ | 1/200 |
| CD3e | FITC | 145-2C11 | BD Pharmingen™ | 1/500 |
| CD3e | Pacific Blue™ | 500A2 | BD Horizon™ | 1/100 |
| CD3e | PE-CF594 | 145-2C11 | BD Horizon™ | 1/500 |
| CD4 | Alexa Fluor® 700 | RM4-5 | BD Pharmingen™ | 1/200 |
| CD4 | PE-Cy™7 | RM4-5 | BD Pharmingen™ | 1/200 |
| CD45 | BV510 | 30/F11 | BD Horizon™ | 1/500 |
| CD45 | PE-CF594 | 30-F11 | BD Horizon™ | 1/500 |
| CD5 | APC | 53-7.3 | BD Pharmingen™ | 1/500 |
| CD64 | BV650 | X45-5/7.1 | BD OptiBuild™ | 1/200 |
| CD8α | APC-H7 | 53-6.7 | BD Pharmingen™ | 1/100 |
| CD8α | BUV737 | 53-6.7 | BD Horizon™ | 1/200 |
| F4/80 | FITC | BM8 | eBiosciences | 1/500 |
| F4/80 | PE | BM8 | BioLegend® | 1/500 |
| FoxP3 | PE | FJK-16S | eBiosciences | 1/200 |
| GATA3 | BUV395 | L50-823 | BD Horizon™ | 1/200 |
| I-A/I-E | BV711 | M5/114.15.2 | BD Horizon™ | 1/500 |
| Ly6C | PerCP-Cy™5.5 | HK1.4 | BioLegend® | 1/200 |
| Ly6G | APC-H7 | 1A8 | BD Pharmingen™ | 1/500 |
| NK1.1 | FITC | PK136 | BD Pharmingen™ | 1/500 |
| NK1.1 | PE | PK136 | BD Pharmingen™ | 1/100 |
| RORγt | Alexa Fluor® 647 | Q31-378 | BD Pharmingen™ | 1/200 |
| Siglec F | PE | E50-2440 | BD Pharmingen™ | 1/100 |
| T-bet | BV786 | O4-46 | BD Horizon™ | 1/200 |
| TCRα | PerCP-Cy™5.5 | B20.1 | BioLegend® | 1/200 |
| TCRβ | Alexa Fluor® 700 | H57-597 | eBiosciences | 1/200 |
| TCR γδ | FITC | GL3 | BD Pharmingen™ | 1/500 |
| TNF | APC | MP6-XT22 | BD Pharmingen™ | 1/200 |
| XCR1 | APC | ZET | BioLegend® | 1/200 |

**Supplementary table 2:** List of antibodies used for flow cytometry.
